# Incidence and Predictors of Surgical Site Infections Following Open Elective and Emergency Surgery: A Prospective Observational Study

**DOI:** 10.7759/cureus.86596

**Published:** 2025-06-23

**Authors:** Keerthy Rajan, Suganya P

**Affiliations:** 1 Department of General Surgery, Sree Balaji Medical College and Hospital, Chennai, IND

**Keywords:** contaminated wounds, diabetes, emergency surgery, infection prevention, multivariate analysis, open surgery, risk factors, surgical site infections (ssi)

## Abstract

Background: Surgical site infections (SSIs) are a major contributor to postoperative morbidity, particularly in low- and middle-income countries, where infection control practices may be less stringent. This study evaluated the incidence and predictors of SSIs in patients undergoing open surgeries at a tertiary hospital in Chennai, India.

Methods: A prospective cohort study was conducted over a two-year period, including 250 patients who underwent open surgeries. Patients were monitored for SSIs from surgery until discharge. Data on patient demographics, comorbidities, lifestyle factors, and surgical variables were collected. Statistical analysis included chi-square tests and multivariate logistic regression to identify independent predictors of SSIs.

Results: The overall incidence of SSIs was 22 (8.8%). Risk factors significantly associated with SSIs were diabetes mellitus 12/73 (16.4%), smoking 5/23 (21.7%), alcohol consumption 6/24 (25.0%), emergency surgery 10/53 (18.9%), and contaminated wounds 9/20 (45.0%). Multivariate logistic regression analysis identified several independent predictors of SSIs. Diabetes mellitus was significantly associated with a higher risk of SSIs (odds ratio, OR: 3.21, 95% CI: 1.41-7.30, p = 0.005), as was undergoing emergency surgery (OR: 2.93, 95% CI: 1.19-7.23, p = 0.020). The presence of contaminated wounds was found to be the strongest predictor, with an OR of 5.82 (95% CI: 2.01-16.87, p = 0.001). Smoking also showed a significant association with increased SSI risk (OR: 2.52, 95% CI: 1.01-6.29, p = 0.048). Additionally, a longer duration of surgery was independently associated with SSIs (OR: 1.86, 95% CI: 1.07-3.21, p = 0.027).

Conclusion: SSIs were associated with diabetes, emergency surgeries, contaminated wounds, smoking, and prolonged surgical duration. These findings may help guide targeted preventive strategies.

## Introduction

Surgical site infections (SSIs) are among the most common complications following surgical procedures, accounting for a substantial portion of healthcare-associated infections worldwide. The burden of SSIs is particularly significant in open surgical procedures, where tissue exposure and operative duration can facilitate microbial contamination [1]. Despite global improvements, SSI rates remain high in low- and middle-income countries due to resource limitations and inconsistent infection control [2].

SSIs prolong hospitalization and increase healthcare costs. Open surgeries are more prone to infection due to prolonged exposure. Emergency surgeries, often performed without optimal preparation, show higher infection rates than elective ones. Comorbidities such as diabetes and smoking impair wound healing. Surgical factors, including wound classification, operative duration, and the level of surgical expertise, are also critical determinants [3]. The objective of this study is to evaluate the incidence of SSIs following open surgeries and to identify significant predictors such as diabetes, smoking, wound contamination, emergency procedures, and prolonged duration of surgery.

## Materials and methods

This prospective observational study was conducted in the Department of General Surgery at Sree Balaji Medical College and Hospital (SBMCH), over a period of 20 months from December 2018 to August 2020, following approval by the Institutional Ethics Committee. The sample size of 250 was estimated based on an expected SSI incidence of 10%, with a 95% confidence interval (CI) and a 5% margin of error, using the standard formula for single proportion studies: n = Z² × P × (1 - P) / d², where Z is the Z-value corresponding to 95% confidence (1.96), P is the estimated incidence (0.10), and d is the margin of error (0.05). This yielded a minimum required sample size of 138. However, to account for potential loss to follow-up and increase statistical power, the sample size was increased to 250 patients. This sample size provided sufficient power for multivariate logistic regression and subgroup analysis.

A total of 250 patients undergoing open surgical procedures, both elective and emergency, were enrolled after obtaining written informed consent. All patients undergoing open elective or emergency abdominal surgeries at SBMCH during the study period were eligible for inclusion in this study. Patients were excluded if they had undergone surgery at the same anatomical site within the previous 30 days, had an active skin infection near the surgical site, were undergoing revision or reoperation procedures, or were pregnant at the time of surgery.

Patients were assessed preoperatively for comorbid conditions, including diabetes mellitus, hypertension, anemia, tuberculosis, and ischemic heart disease. All patients underwent standard preoperative preparation, including shaving the operative site, receiving tetanus toxoid, and prophylactic administration of antibiotics 30 minutes prior to incision. Povidone-iodine scrub was used intraoperatively for site preparation.

SSIs were classified based on the Centers for Disease Control and Prevention (CDC)/National Healthcare Safety Network (NHSN) guidelines into superficial incisional, deep incisional, and organ/space infections. Wound classes were defined as clean, clean-contaminated, contaminated, and dirty, according to the CDC surgical wound classification.

Surveillance for SSIs was conducted daily during inpatient stay until postoperative day (POD) 5 or discharge, and patients were followed up after discharge for 30 days, either through in-person surgical outpatient visits or telephonic review for those unable to return. Any signs of infection identified after discharge were reviewed and recorded by the surgical team using the same CDC/NHSN criteria. All patients received a single dose of intravenous (IV) ceftriaxone 1 g and metronidazole 500 mg, administered 30 minutes prior to surgical incision. In patients with known beta-lactam allergy, ciprofloxacin 400 mg IV was used as an alternative.

Surgery duration was categorized as less than one hour, one to two hours, and greater than two hours for univariate analysis. However, for multivariate logistic regression, duration was included as a binary variable (greater than two hours vs. less than or equal to two hours) based on its distribution and clinical relevance. Statistical analysis was performed using IBM Statistical Package for the Social Sciences (SPSS) Statistics for Windows, version 26.0 (IBM Inc., Armonk, NY). Variable inclusion in the multivariate logistic regression was based on p < 0.1 in univariate analysis, and backward stepwise elimination was used for model refinement. No imputation was used, and records with missing critical data points were excluded pairwise from specific analyses. The overall rate of missing data was minimal (<2%).

To ensure detection of late-onset SSIs, all patients were followed for 30 days postoperatively. Follow-up was done either in person at scheduled postoperative visits or, if the patient could not return, via structured telephonic follow-up by the surgical team. Any reported wound-related symptoms triggered an in-clinic review or referral for evaluation. This process ensured standardized postdischarge surveillance.

Data collected included demographic information, type and duration of surgery, wound classification (clean, clean-contaminated, or contaminated), and the identity of the operating surgeon. Postoperatively, wounds were examined daily, with routine dressing changes up to the fifth POD. Any suspected infection was assessed both clinically and through microbiological testing. Wound swabs were cultured, and appropriate antibiotics were administered based on sensitivity patterns. The wound healing process and need for secondary suturing were also recorded (Table 1).

**Table 1:** Summary of study protocol and methods TT: tetanus toxoid; POD: postoperative day; SSI: surgical site infection

Component	Description
Study design	Prospective observational study
Duration	December 2018 to August 2020 (20 months)
Setting	Department of General Surgery, Sree Balaji Medical College and Hospital
Sample size	250 patients
Inclusion criteria	Patients undergoing elective or emergency open surgeries
Exclusion criteria	Previous surgery, skin infections, and recurrent surgeries
Preoperative protocol	Site shaving, TT injection, test dose of Xylocaine, and prophylactic antibiotics
Intraoperative protocol	Povidone-iodine scrub and sterile field maintenance
Postoperative monitoring	Daily wound exam; POD 2-5 follow-up; culture testing as needed
Microbiology	Swab cultures and sensitivity testing
Antibiotic treatment	Empirical followed by sensitivity-guided therapy
Outcome measures	SSI occurrence, organism type, and treatment outcome

Data were analyzed using SPSS version 26.0. Categorical variables were analyzed using chi-square tests. Independent t-tests were used for continuous variables. Variables with p < 0.1 in univariate analysis were entered into multivariate logistic regression using backward stepwise elimination.

## Results

Of the 250 patients enrolled, 22 (8.8%) developed SSIs. A higher incidence of SSIs was observed among patients with diabetes mellitus (16.4%), smokers (21.7%), and those who consumed alcohol (25%). Emergency surgeries had a significantly greater infection rate (18.9%) compared to elective procedures (6.1%). Contaminated wounds showed the highest infection rate at 45%, while clean and clean-contaminated wounds had lower rates. Procedures lasting over two hours had a 17.1% infection rate, compared to 4.2% in surgeries between one and two hours. Multivariate logistic regression confirmed diabetes, contaminated wounds, emergency surgery, smoking, and prolonged duration as independent predictors of SSIs. These findings are further detailed in Table 2.

**Table 2:** Distribution of demographic and clinical characteristics among study participants

Variable	Category	Frequency (n)	Percentage (%)
Age group (years)	≤20	0	0
	21-30	64	25.6
	31-40	100	40
	41-50	45	18
	51-60	39	15.6
	≥61	2	0.8
	Total	250	100
Gender	Female	113	45.2
	Male	137	54.8
	Total	250	100
General risk factors	Diabetes mellitus	73	29.2
	Hypertension	62	24.8
	Anemia	49	19.6
	Smokers	23	9.2
	Alcoholic	24	9.6
	Tuberculosis	6	2.4
Nature of the surgery	Elective	197	78.8
	Emergency	53	21.2
	Total	250	100
Type of surgery (groupwise)	Group 1	91	36.4
	Group 2	60	24
	Group 3	17	6.8
	Group 4	23	9.2
	Group 5	41	16.4
	Group 6	18	7.2
	Total	250	100
Wound classification	Clean	174	69.6
	Clean contaminated	56	22.4
	Contaminated	20	8
	Total	250	100
Surgeon performing surgery	Chief	29	11.6
	Assistant	62	24.8
	Postgraduate	140	56
	Intern	19	7.6
	Total	250	100
Duration of surgery	<1 hour	36	14.4
	1-2 hours	144	57.6
	>2 hours	70	28
	Total	250	100
Postoperative wound status	Infected	22	8.8
	Not infected	228	91.2
	Total	250	100

Table 3 presents a comparative analysis of patients with and without postoperative wound infections, applying appropriate statistical methods. The mean age of patients who developed wound infections was higher (42.5 years) than that of those who did not (38.36 years), with the difference nearing statistical significance (p = 0.053). Gender did not show a significant association with wound infection status (p = 0.672), and similar nonsignificant results were observed for hypertension (p = 0.425), tuberculosis (p = 0.491), anemia (p = 0.699), and the level of the surgeon performing the procedure (p = 0.523). In contrast, several variables demonstrated a statistically significant association with increased risk of postoperative wound infection. Patients with diabetes mellitus had a markedly higher infection rate (16.4%) compared to nondiabetics (5.6%), with the difference being statistically significant (p = 0.006). Lifestyle-related factors such as smoking and alcohol use were also significantly associated with infection, with smokers and alcohol users exhibiting infection rates of 21.7% and 25.0%, respectively (p = 0.022 and p = 0.030). Emergency surgeries were found to have a significantly higher infection rate (18.9%) compared to elective procedures (6.1%, p = 0.004).

**Table 3:** Comparison of postoperative surgical wound infection with various risk factors

Variable	Category	Infected (n)	Not infected (n)	Total (n)	p value	Statistical test
Mean age	-	42.5 years	38.36 years	-	0.053	Independent t-test
Gender	Female	9 (8%)	104 (92%)	113	0.672	Chi-square test
	Male	13 (9.5%)	124 (90.5%)	137		
Hypertension	Present	7 (11.3%)	55 (88.7%)	62	0.425	Chi-square test
	Absent	15 (8%)	173 (92%)	188		
Diabetes mellitus	Present	12 (16.4%)	61 (83.6%)	73	0.006	Significant chi-square test
	Absent	10 (5.6%)	167 (94.4%)	177		
Tuberculosis	Present	1 (16.7%)	5 (83.3%)	6	0.491	Chi-square test
	Absent	21 (8.6%)	223 (91.4%)	244		
Anemia	Present	5 (10.2%)	44 (89.8%)	49	0.699	Chi-square test
	Absent	17 (8.5%)	184 (91.5%)	201		
Smoking	Present	5 (21.7%)	18 (78.3%)	23	0.022	Significant chi-square test
	Absent	17 (7.5%)	210 (92.5%)	227		
Alcohol use	Present	6 (25%)	18 (75%)	24	0.030	Significant chi-square test
	Absent	16 (7.1%)	210 (92.9%)	226		
Nature of surgery	Elective	12 (6.1%)	185 (93.9%)	197	0.004	Significant, chi-square test
	Emergency	10 (18.9%)	43 (81.1%)	53		
Wound class	Clean	7 (4%)	167 (96%)	174	<0.001	Significant chi-square test
	Clean contaminated	6 (10.7%)	50 (89.3%)	56		
	Contaminated	9 (45%)	11 (55%)	20		
Surgery performed by	Chief	3 (10.3%)	26 (89.7%)	29	0.523	Chi-square test
	Assistant professor	7 (11.3%)	55 (88.7%)	62		
	Postgraduate	10 (7.1%)	130 (92.9%)	140		
	Intern	2 (10.5%)	17 (89.5%)	19		
Duration of surgery	<1 hour	4 (11.1%)	32 (88.9%)	36	0.006	Significant chi-square test
	1-2 hours	6 (4.2%)	138 (95.8%)	144		
	>2 hours	12 (17.1%)	58 (82.9%)	70		

Wound classification showed a strong correlation with infection rates; contaminated wounds had the highest rate of infection (45%), followed by clean-contaminated (10.7%) and clean wounds (4.0%), with a p value of <0.001. Duration of surgery was another significant factor, as procedures lasting more than two hours were associated with a higher infection rate (17.1%) compared to shorter surgeries (p = 0.006). These findings underscore the influence of patient comorbidities, behavioral risk factors, surgical urgency, wound contamination level, and operative duration on the incidence of postoperative wound infections.

Multivariate logistic regression was performed for variables with p < 0.1 in univariate analysis. Diabetes mellitus, emergency surgery, contaminated wounds, smoking, and operative duration greater than two hours were identified as independent predictors of SSI. These results are detailed in Table 4.

**Table 4:** Multivariate logistic regression analysis of predictors of SSI SSI: surgical site infection

Predictor	Odds ratio	95% confidence interval	p value
Diabetes mellitus	3.21	1.41-7.30	0.005
Emergency surgery	2.93	1.19-7.23	0.020
Contaminated wound	5.82	2.01-16.87	0.001
Smoking	2.52	1.01-6.29	0.048
Duration >2 hours	1.86	1.07-3.21	0.027

Table 5 outlines the clinical and microbiological characteristics, treatment response, and outcomes of postoperative wound infections in the study. Most infections were detected early, with 45.5% diagnosed on POD 2, 40.9% on POD 3, and 13.6% on POD 4. Detection was achieved primarily through a combination of clinical evaluation and microbiological testing (90.9%), with a small proportion (9.1%) based on clinical signs alone. Among the 20 culture-positive cases, Staphylococcus spp. was the most frequently isolated organism (40%), followed by *Escherichia coli* (25%), Streptococcus spp. (20%), and Pseudomonas spp. (10%), while other organisms accounted for 5%. Antibiotic sensitivity testing revealed that Linezolid was the most effective drug (35%), followed by meropenem and cefotaxime (20% each), with piperacillin-tazobactam and amikacin effective in 10% of cases, and amoxiclav in 5%. Treatment outcomes were favorable in most cases, with 90.9% of infections resolving and only 9.1% persisting. Secondary suturing was required in 90.9% of the infected cases, reflecting a high need for further surgical wound management.

**Table 5:** Detailed analysis of postoperative wound infections POD: postoperative day

Variable	Category	Frequency (n)	Percentage (%)
Day of detection	POD 2	10	45.5
	POD 3	9	40.9
	POD 4	3	13.6
Method of detection	Clinical + microbiology	20	90.9
	Clinical only	2	9.1
Organism grown (n = 20)	Staphylococcus spp.	8	40
	E. coli	5	25
	Streptococcus spp.	4	20
	Pseudomonas spp.	2	10
	Others	1	5
Antibiotic sensitivity (n = 20)	Linezolid	7	35
	Meropenem	4	20
	Taxim (cefotaxime)	4	20
	Piptaz (piperacillin + tazobactam)	2	10
	Amikacin	2	10
	Amoxiclav	1	5
Outcome after treatment	Resolved	20	90.9
	Not resolved	2	9.1
Secondary suturing required	Done	20	90.9
	Not done	2	9.1

## Discussion

The overall SSI rate was 8.8%, consistent with global reports. Alkaaki et al. [1] reported similar incidence rates in a prospective cohort study from Canada, highlighting the global relevance of SSI risk in abdominal surgeries. Mawalla et al. [2], in a study conducted in Tanzania, also demonstrated high SSI rates, particularly in emergency and contaminated wound categories, findings that reinforce our results. Several significant risk factors for SSI emerged in our analysis, including diabetes mellitus, prolonged surgical duration, emergency surgery, and wound contamination. Blumetti et al. [3] emphasized that SSI risk varies with the type and location of surgery, as well as host and procedural factors. In our study, contaminated wounds showed a significantly higher infection rate of nine out of 20 (45%), aligning with results by Acín-Gándara et al. [4], who documented elevated SSI rates in colon surgeries, especially in patients with Class III or IV wounds.

Surgical procedures were categorized into six groups based on the institutional classification system, which reflects the anatomical and procedural nature of the operations performed. Group 1 included gastrointestinal surgeries such as appendectomy, hernia repair, and bowel resections. Group 2 comprised hepatobiliary and pancreatic procedures like cholecystectomy and liver resections. Group 3 encompassed urological surgeries, including nephrectomy and transurethral resection of the prostate. Group 4 covered breast and endocrine surgeries such as mastectomy and thyroidectomy. Group 5 consisted of emergency laparotomies and trauma-related procedures. Group 6 included miscellaneous surgeries such as skin and soft tissue procedures and vascular access surgeries. This classification allowed for a structured analysis of surgical type in relation to postoperative outcomes [2,3].

The reported SSI incidence of 8.8% reflects infections identified during hospitalization only. As postdischarge surveillance was limited to outpatient or telephonic follow-up, late-onset SSIs may have been underdiagnosed. Therefore, the true 30-day incidence could be marginally higher than reported. From a preventive standpoint, early administration of antibiotics and maintenance of sterile technique are crucial. Dellinger [5] stressed the importance of infection control protocols, including timely prophylaxis and operative field preparation, which we ensured in our protocol. Additionally, the microbiological profile in our study, dominated by Staphylococcus spp. and E. coli, matches findings by Narula et al. [6], who highlighted similar organisms in Indian tertiary centers. Kirby and Mazuski [7] reviewed SSI prevention strategies and identified modifiable intraoperative practices that reduce contamination and improve patient outcomes. Their recommendations support our findings that procedures lasting more than two hours are associated with higher infection rates, 12 out of 70 (17.1%) in our cohort. Our data also corroborate WHO guidelines by Allegranzi et al. [8], which showed that longer operative time and poor wound classification are key determinants of infection.

Historical surveillance studies, such as Khairy et al. [9] and Segal et al. [10], underline that despite technological advances, SSI rates have remained stubbornly high in certain contexts, particularly in public hospitals and teaching institutions. This may relate to case complexity and surgeon experience, although our findings did not show a statistically significant difference based on the operating surgeon’s rank. Geubbels et al. [11], through national surveillance systems in the Netherlands, demonstrated the utility of continuous monitoring, which could help maintain low SSI rates through targeted interventions. Culver et al. [12] further developed the patient risk index, which supports stratification based on factors such as wound class and comorbidities, principles we applied in our analysis. The association between systemic health factors and SSI is further supported by statistical models such as those detailed by de Oliveira et al. [13], who emphasized the importance of multivariable assessment in infection epidemiology. This is echoed in Moro et al.’s [14] findings from Italian hospitals, where wound infection rates were substantially influenced by duration and contamination, in line with our results.

Smoking and alcohol use were consistent risk factors, as shown in earlier studies. Mawalla et al. [2] also identified these habits as contributors to impaired healing and increased SSI risk, particularly in settings with limited preoperative optimization. Similarly, Khairy et al. [9] documented that patient-related comorbidities significantly influenced infection rates in a large U.S. cohort, reinforcing the critical importance of individualized perioperative care. The microbiological spectrum in our setting mirrors that reported by Narula et al. [6], where Staphylococcus spp. and *E. coli* were the most common isolates, pointing toward a consistent pattern of community- and hospital-acquired pathogens in surgical infections.

Finally, Korol et al. [15] posed the question of surgical expertise impacting infection risk. Higher infection rates in senior-led cases likely reflect case complexity, not surgeon skill, as shown in Table 6. Despite the strengths of this prospective study design and comprehensive variable analysis, several limitations must be acknowledged. The single-center nature of the study may limit the generalizability of the findings to other institutions with differing surgical practices and patient populations. The sample size, while adequate for preliminary assessment, may not capture the full spectrum of risk factor interactions. Additionally, the follow-up period was limited to in-hospital stay, and any delayed SSIs presenting after discharge may have been missed. Microbiological evaluation was performed only in clinically suspected cases, possibly underestimating subclinical infections. Future multicentric studies with larger cohorts and extended postoperative surveillance would be valuable in validating and expanding upon these findings.

**Table 6:** Comparative analysis of SSI risk factors with literature evidence SSI: surgical site infection; POD: postoperative day

Risk factor/finding	Our study observation	Supporting literature
Overall SSI incidence	8.8%	Alkaaki et al. [1] and Mawalla et al. [2]
Emergency surgery	Higher infection rate (18.9%) vs. elective (6.1%)	Mawalla et al. [2] and Korol et al. [15]
Wound classification (contaminated)	45% SSI in contaminated wounds	Acín-Gándara et al. [4] and Culver et al. [12]
Diabetes mellitus	Strongly associated with SSI (p = 0.006)	Mawalla et al. [2] and Khairy et al. [9]
Operative duration >2 hours	Significant increase in SSI incidence (p = 0.006)	Kirby and Mazuski [7], Allegranzi et al. [8], and Moro et al. [14]
Staphylococcus spp. and *E. coli*	Most common organisms	Narula et al. [6] and Khairy et al. [9]
Microbiological diagnosis	91% confirmed via culture; Linezolid most sensitive	Narula et al. [6], and Kirby and Mazuski [7]
Postgraduate-led surgeries	The majority were performed by postgraduates (56%); no significant SSI difference by surgeon level	Korol et al. [15] and Alkaaki et al. [1]
Antibiotic prophylaxis	Given 30-minute preop; effective when used appropriately	Dellinger [5] and Segal et al. [10]
Need for surveillance	High rate of detection by POD 2; useful for early treatment	Geubbels et al. [11] and de Oliveira et al. [13]

## Conclusions

In conclusion, this prospective observational study identified an SSI rate of 8.8% following open surgical procedures, with diabetes mellitus, emergency surgery, contaminated wounds, smoking, and prolonged operative duration emerging as significant independent predictors. These results highlight the critical role of both patient-related comorbidities and intraoperative factors in SSI development. The findings advocate for the integration of structured risk stratification protocols into surgical planning, with particular attention to modifiable factors. Implementation of timely prophylactic measures, stringent aseptic techniques, and close postoperative surveillance in high-risk patients may help reduce SSI incidence and improve clinical outcomes. Given the study’s setting in a tertiary care center within a resource-limited context, it also provides important regional insights that contribute to the broader understanding of SSI prevention in low- and middle-income countries.
